# Correlation between circulating dephosphorylated uncarboxylated matrix Gla protein and vascular calcification in peritoneal dialysis patients

**DOI:** 10.1177/03913988241293980

**Published:** 2024-10-31

**Authors:** Liman Mao, Haole Huang, Meiyang Zhou, Canxin Zhou

**Affiliations:** Department of Nephrology, The Affiliated People’s Hospital, Ningbo University, Zhejiang, China

**Keywords:** Peritoneal dialysis, vascular calcification, dephosphorylated uncarboxylated matrix Gla protein, abdominal aortic calcification

## Abstract

**Introduction:**

To explore the association between serum Dephosphorylated uncarboxylated matrix Gla protein (dp-ucMGP) and abdominal aortic calcification (AAC) in peritoneal dialysis (PD) patients.

**Methods:**

A total 128 PD patients and 120 healthy controls were enrolled into the study. Serum dp-ucMGP was measured by enzyme-linked immunosorbent assay. Abdominal lateral plain radiography was used to evaluate the abdominal aortic calcification score (AACS). PD patients were divided into two groups according to the presence or absence of AAC. The relationships between dp-ucMGP levels and AACS were assessed by Spearman analysis and the value of dp-ucMGP in predicting AAC was evaluated by receiver operating characteristic (ROC).

**Results:**

Serum dp-ucMGP in PD patients were significantly higher than controls (*p* < 0.05). And PD patients with AAC had higher serum dp-ucMGP than that of PD patients without AAC (*p* < 0.05). The serum dp-ucMGP levels was positively associated with AACS (*r* = 0.794, *p* < 0.0001) in PD patients. The multivariate logistic regression analyses showed that serum dp-ucMGP was independent factors of AAC in PD patients (OR = 2.555, 95% CI = 1.415–4.609). The area under ROC curve of dp-ucMGP was 0.9227, the corresponding sensitivity was 0.86, and the specificity was 0.92.

**Conclusion:**

Serum dp-ucMGP levels were positively associated with the AACS in PD patients. Higher serum dp-ucMGP level is independently associated with AAC in PD patients.

## Introduction

Patients with chronic kidney disease (CKD), especially patients with end-stage renal disease (ESRD), exhibit higher mortality and incidence of cardiovascular disease (CVD) compared with the non-CKD cohort, accounting for more than 50% of all deaths in CKD patients.^1–3^ Accumulating evidence suggests that vascular calcification (VC) is an important factor associated with CVD and increases the all-cause mortality in patients with ESRD.^2–4^ Therefore, it is important to find out potential biomarkers for early detection of patients prone to VC, allowing for more targeted intervention to improve the cardiovascular outcome of patients on dialysis.

Matrix Gla protein (MGP), a 12 kDa vitamin K-dependent protein, secreted by cartilage and arterial wall cells, which is considered as a potent inhibitor of VC via scavenging free, reactive calcium ions, phosphorus ions, and hydroxyapatite crystals from the arterial wall.^5–7^ Dephosphorylated uncarboxylated matrix Gla protein (dp-ucMGP) represents the completely non-functional non-phosphorylated and uncarboxylated form of MGP, which is set free into the circulation because of the low affinity for calcium and matrix vesicles.^8,9^ In vitro and in vivo studies has coherently detected dp-ucMGP is accumulated in calcified vessels and circulating dp-ucMGP is a strong indicator of vitamin K status, which plays a pivotal role in the pathogenesis of VC. Moreover, observational studies reported that dp-ucMGP was independently associated with various indicators of VC both in general population and cohorts with diabetes, atherosclerotic disease, heart failure, and CKD.^8,10–13^ In contrast with these studies, Schlieper et al. and other studies reported no association between circulating dpucMGP and VC in cohorts of CKD or hemodialysis (HD) patients.^14–16^ Additionally, the data regarding the direct association of dp-ucMGP levels and VC in peritoneal dialysis (PD) patients remains very limited.

In this study, we measured serum dp-ucMGP levels and compared the clinical characteristics in a cohort of PD patients with or without abdominal aortic calcification (AAC). The association of serum dp-ucMGP levels and the presence of vascular calcification in PD patients was also investigated in our study.

## Methods

### Study population

**Figure 1.**
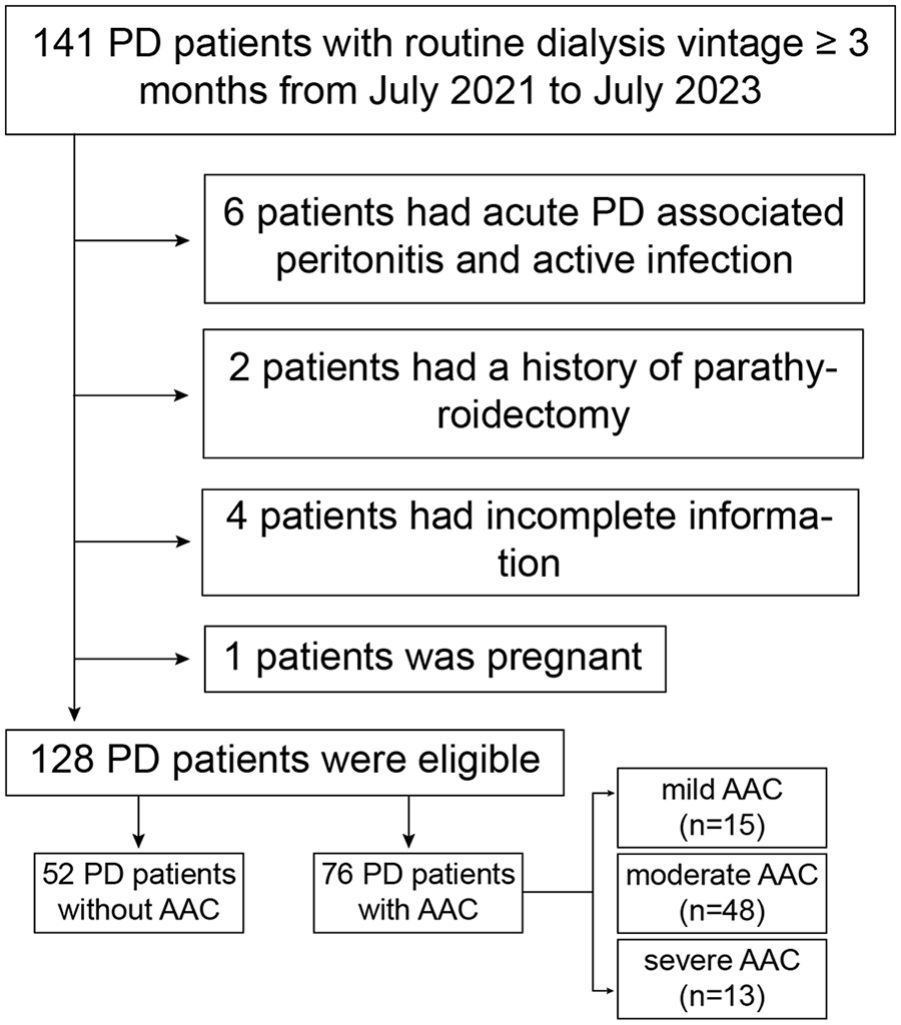
Flowchart of this study. PD: peritoneal dialysis; AAC: abdominal aortic calcification.

Eligible PD patients in Department of Nephrology of the Affiliated People’s Hospital of Ningbo University from July 2021 to July 2023 were enrolled. All selected patients were treated with Baxter’s dual bag glucose dialysate from China for PD. The details of PD were available in Supplemental Material. Inclusion criteria were undergoing PD already for 3 months with a routine dialysis schedule and age ranged from 19 to 75 years. Exclusion criteria included inability to provide informed consent, acute PD associated peritonitis and active infection, pregnant women, chronic rheumatic heart disease, congenital heart disease, active malignancy, liver cirrhosis, systemic lupus erythematosus, history of undergoing long-term hemodialysis, and history of kidney transplantation or parathyroidectomy. To compare dp-ucMGP levels between controls and PD patients, healthy volunteers were recruited, matched with the age and gender of the investigated group. The subject was excluded from the control group if he had history of proteinuria, hematuria, decreased glomerular filtration rate, or AAC. The protocol of our study was in accordance with the Helsinki Declaration of Human Rights and was approved by the Ethics Committee of the Affiliated People’s Hospital of Ningbo University. And all selected patients signed informed consent forms (Figure 1).

### Clinical and biochemical evaluation

The general information of the selected cases including age, gender, body mass index (BMI, kg/m^2^), dialysis age (months), hypertension n (%), diabetes n (%), smoking status and medication data were recorded. Data were extracted from electronic medical files and completed through interviews with the patients.

The following laboratory data were collected: calcium (Ca), phosphate (P), hemoglobin (Hb), albumin (ALB), alkaline phosphatase (ALP), high sensitivity C reaction protein (hs-CRP), intact parathormone (iPTH), 25-hydroxyvitamin D (25-(OH)D), uric acid (UA), creatinine (Cr), β2-microglobulin (β2-MG), fasting blood glucose (FBG), type A1C glycosylated hemoglobin (HbA1c), and lipids profile in serum. The corrected blood calcium was calculated by: measured blood calcium (mmol/L) + [40-blood albumin (g/L)] × 0.025. The product of calcium and phosphorus [Ca-P product (mmol^2^/L^2^)] = corrected blood calcium (mmol/L) × blood phosphorus (mmol/L). Fractional urea clearance (Kt/V urea) was calculated by the Daugirdas formula. Residual renal function was calculated as an average of 24-h urinary urea and creatinine clearance.^17^

### Assessment of serum dp-ucMGP

Serum dp-ucMGP levels were determined using the commercially available Enzyme-linked immunosorbent assay (ELISA) kit (Fine Test, Wuhan, China) according to the manufacturer’s instructions.^18^ Details and descriptions of ELISA is shown in the Supplemental Material.

### Abdominal aortic calcification score (AACS)

AACS was assessed by two radiologists with senior professional titles using Kauppila scoring,^19^ and the average of all scores was taken as the patient’s final AACS. AACS ranged from 0 to 24 points, and the severity of calcification was classified into three groups based on the AACS: no or mild calcification group (AACS ⩽ 4), moderate calcification group (5 ⩽ AACS ⩽ 15), and severe calcification group (AACS ⩾ 16). Detailed information about AACS is provided in Supplemental Material.

### Statistical analysis

Normal distribution data were expressed as mean ± standard deviation (SD), and the comparison between two groups was conducted using independent sample *t*-test. Non-normal distribution data were expressed represented by median (Q25 and Q75), and Mann Whitney *U* test was used for comparison between two groups. Categorical variables were presented as frequencies (percentages), and the χ^2^ test was used to compare categorical variables. Statistical significance was set at the level of *p* < 0.05. The correlation between clinical data with bivariate normal distribution is analyzed using Pearson test, otherwise Spearman correlation analysis is used. Multivariate logistic regression to analyze the independent influencing factors of AAC in PD patients.

Multivariate linear and logistic regression analyses were performed to identify independent determinants of dp-ucMGP and presence of VC, respectively. Continuous variables were transformed to one standard deviation (1-SD) increments in linear and logistic regression analyses. Variables with statistical significance in the Spearman correlation analyses were first entered into a multivariate regression model and were then eliminated with a backward-selection estimation, where variables with *p* > 0.05 were eligible for removal from the model.

Receiver operation characteristic (ROC) curves were used to determine optimal cutoff values of dp-ucMGP by maximizing the Youden index (sensitivity + specificity − 1). All statistical analyses were performed using the statistical package 22.0 (IBM, Armonk, NY, USA). Figures were created using GraphPad Prism version 8.3.1 (GraphPad Software, San Diego, USA).

## Results

### Clinical and biochemical characteristics of study population

The clinical and biochemical characteristics of our study participants were shown in Table 1. Our study enrolled 128 PD patients (66 male patients, mean age 60.2 ± 12.4 years, average PD duration 28.4 ± 9.7 months) and 120 healthy controls. Patients and controls were comparable in age, sex ratio, and BMI. Compared with healthy controls, the patients with PD had higher levels of LDL, corrected Ca, P, Ca-P product, ALP, iPTH, Cr, UA, hs-CRP, and dp-ucMGP. Additionally, the levels of Hb, ALB, HDL, and 25(OH)D were lower in the PD group than those in control group. The proportion of participants with hypertension, T2DM, and smoking habit of PD patients were higher than those of controls.

**Table 1.** Comparisons of clinical and biochemical characteristics between the controls and PD patients.

Parameters	Control (*n* = 120)	PD patients (*n* = 128)	*p*
Age, year	56.7 ± 9.9	60.2 ± 12.4	0.062
Male *n*, (%)	61 (50.83)	66 (51.56)	0.909
BMI, kg/m^2^	21.71 ± 3.42	22.19 ± 3.83	0.416
Dialysis vintage, month	NA	28.4 ± 9.7	–
HBP *n*, (%)	11 (9.17)	121 (94.53)	<0.001
T2DM *n*, (%)	0	26 (20.31)	<0.001
Smoking habit *n*, (%)	9 (7.50)	23 (17.97)	0.002
Use of calcium carbonate *n*, (%)	NA	58 (45.3)	–
Use of non-calcium-containing phosphate binder *n*, (%)	NA	51 (39.8)	–
Use of calcitriol *n*, (%)	NA	45 (35.2)	–
Hb, g/L	129.23 ± 12.46	111.84 ± 12.35	<0.001
ALB g/L	43.19 ± 4.17	35.26 ± 4.22	<0.001
TG, mmol/L	1.5 (1.0, 2.8)	1.7 (1.1, 2.7)	0.15
TC, mmol/L	3.75 ± 0.94	3.56 ± 0.73	0.163
HDL, mmol/L	1.4 (1.0, 1.5)	1.1 (0.9, 1.6)	0.042
LDL, mmol/L	2.17 ± 0.48	2.85 ± 0.61	0.037
FBG, mmol/L	5.48 ± 1.63	6.97 ± 2.15	0.527
HbA1c	5.23 ± 1.25	6.19 ± 1.51	0.069
Corrected Ca, mmol/L	2.2(2.1, 2.5)	2.4 (2.0, 2.6)	0.047
P, mmol/L	1.24 ± 0.33	1.71 ± 0.42	<0.001
Ca-P product, mmol^2^/L^2^	2.4(2.1, 3.2)	3.2 (2.2, 5.1)	0.041
25(OH)D, µg/L	32.65 ± 5.36	18.51 ± 4.61	<0.001
ALP, U/L	104.5 (74.7, 127.75)	182.1 (89.5, 272.5)	<0.001
iPTH, ng/L	175.0 (56.4, 317.4)	258.4 (151.2, 424.1)	<0.001
Cr, µmol/L	75.27 ± 9.27	871.5 ± 296.1	<0.001
β2-MG, mg/L	NA	27.82 ± 4.74	–
UA, µmol/L	317 ± 70.19	425 ± 85.27	<0.001
hs-CRP, mg/L	1.5 (0.9, 3.4)	6.4 (3.2, 15.1)	<0.001
Kt/V urea, per week	NA	1.9 (1.7, 2.1)	–
RRF, mL/min	NA	3.05 (0.61, 5.86)	–
dp-ucMGP, pmol/L	427.2 ± 44.3	578.4 (530.4, 632.6)	<0.001
AACS	NA	4 (0, 13)	–

Conversion factor for units: dp-ucMGP in pg/mL to pmol/L, *80.952. NA means not assessed. PD: peritoneal dialysis; BMI: body mass index; HBP: hypertension; T2DM: type 2 diabetes mellitus; Hb: hemoglobin; ALB: albumin; TG: triglyceride; TC: total cholesterol; HDL: high-density lipoprotein; LDL: low-density lipoprotein; FBG: fasting blood glucose; HbA1c: type A1C glycosylated hemoglobin; Ca: calcium; P: phosphate; 25(OH)D: 25-hydroxyvitamin D; ALP: alkaline phosphatase; iPTH: intact parathyroid hormone; Cr: creatinine; β2-MG: β2-microglobulin; UA: uric acid; hs-CRP: high sensitivity C reaction protein; Kt/V urea: fractional urea clearance; RRF: residual renal function; dp-ucMGP: dephosphorylated uncarboxylated matrix Gla protein; AACS: abdominal aortic calcification score.

### Comparisons of clinical and biochemical characteristics between PD patients with or without AAC

We divided PD patients into two groups according to the presence or absence of AAC. Seventy-six patients (59.4%) were found to have AAC. As shown in Table 2, PD patients with AAC had higher serum dp-ucMGP [620.3 (591.8, 668.3) pmol/L versus 529.0 (509.8, 553.9) pmol/L, *p* < 0.001] than that of PD patients without AAC. BMI, PD vintage, and the proportion of smoking of the PD-AAC group were higher than those of PD-nonAAC group. Moreover, the PD-AAC group had higher serum corrected Ca, P, Ca-P product, iPTH, UA, and hs-CRP than the PD-nonAAC group. Serum TG and ALP of the PD-AAC group tended to be higher than that of PD-nonAAC group, but the difference was not significant. However, PD patients with AAC had lower serum ALB and RRF than the PD-nonAAC group.

**Table 2.** Comparisons of clinical and biochemical characteristics between PD patients with or without AAC.

Parameters	Non-AAC (*n* = 52)	AAC (*n* = 76)	*p*
Age, year	59.4 ± 10.6	62.4 ± 12.2	0.074
Male *n*, (%)	27 (51.92)	42 (55.26)	0.710
BMI, kg/m^2^	21.17 ± 3.29	22.63 ± 3.74	0.031
Dialysis vintage, month	14.6 ± 4.7	35.5 ± 11.3	<0.001
HBP *n*, (%)	48 (92.31)	73 (96.05)	0.360
T2DM *n*, (%)	11 (21.15)	15 (19.74)	0.845
Smoking habit *n*, (%)	4 (7.69)	19 (25.00)	0.012
Use of calcium carbonate *n*, (%)	22 (42.3)	36 (47.4)	0.593
Use of non-calcium-containing phosphate binder *n*, (%)	19 (36.5)	32 (42.1)	0.584
Use of calcitriol *n*, (%)	17 (32.7)	28 (36.8)	0.708
TG, mmol/L	1.8 (1.1, 2.6)	2.1 (1.4, 2.9)	0.072
TC, mmol/L	3.43 ± 0.86	3.51 ± 0.92	0.616
HDL, mmol/L	1.3 (0.9, 1.6)	1.1 (0.9, 1.4)	0.442
LDL, mmol/L	2.19 ± 0.35	2.23 ± 0.51	0.537
FBG, mmol/L	6.38 ± 1.83	6.88 ± 2.05	0.612
HbA1c	6.23 ± 1.05	6.35 ± 1.24	0.429
Corrected Ca, mmol/L	1.6 (1.1, 2.6)	2.3 (1.5, 4.5)	0.034
P, mmol/L	1.84 ± 0.35	1.97 ± 0.46	<0.001
Ca-P product, mmol^2^/L^2^	2.9 (2.6, 3.8)	3.4 (2.9, 4.2)	0.016
25(OH)D, µg/L	19.36 ± 4.73	19.25 ± 4.57	0.628
ALP, U/L	96.50 (79.75, 130.25)	113.50 (100.25, 131.25)	0.054
iPTH, ng/L	182.6 (60.7, 263.4)	384.8 (242.5, 653.1)	<0.001
Cr, µmol/L	910.8 ± 316.9	836.0 ± 274.9	0.382
β2-MG, mg/L	25.18 ± 4.54	26.74 ± 4.67	0.315
UA, µmol/L	363.52 ± 67.39	431.75 ± 72.57	<0.001
hs-CRP, mg/L	6.2 (1.3, 14.5)	7.0 (2.9, 13.9)	<0.01
Kt/V urea, per week	1.8 (1.7, 2.1)	1.9 (1.7, 2.3)	0.541
RRF, mL/min	2.79 (0.31, 5.17)	1.53 (0.18, 3.91)	<0.001
dp-ucMGP, pmol/L	529.0 (509.8, 553.9)	620.3 (591.8, 668.3)	<0.001
AACS	0	12 (6, 17)	<0.001

AAC: abdominal aortic calcification. Other abbreviations are as defined in Table 1.

### Comparison of serum dp-ucMGP levels between controls and PD patients

**Figure 2.**
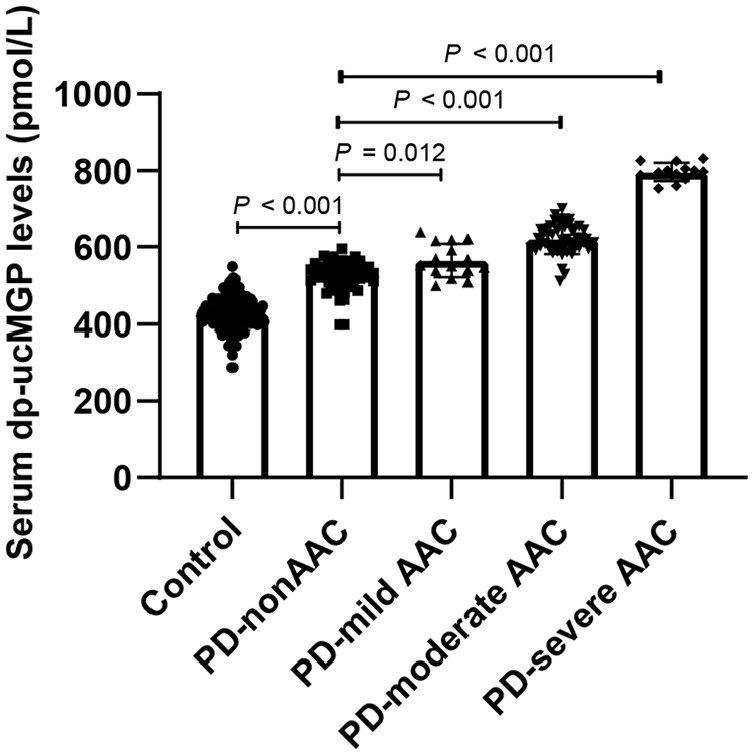
Comparisons of serum dp-ucMGP among different groups presented as a dot-plot.

According to the AACS, we divided PD-AAC patients into three groups. There were 15 (19.7%) patients in the mild calcification group, 48 (63.2%) patients in the moderate calcification group, and 13 (17.1%) patients in the severe calcification group. The levels of serum dp-ucMGP were 427.2 ± 44.3 pmol/L in the controls, 529.0 (509.8, 553.9) pmol/L in the PD-nonAAC group, 564.5 ± 43.6 pmol/L in the PD-mild AAC group, 620.5 ± 39.0 pmol/L in the PD-moderate AAC group, and 795.8 ± 23.6 pmol/L in the PD-severe AAC group. As shown in Figure 2, the more severe the vascular calcification, the higher the serum dp-ucMGP level, which suggested that dp-ucMGP has a positive correlation with the severity of vascular calcification in PD patients.

### Correlation analysis of serum dp-ucMGP, AACS, and clinical parameters

**Figure 3.**
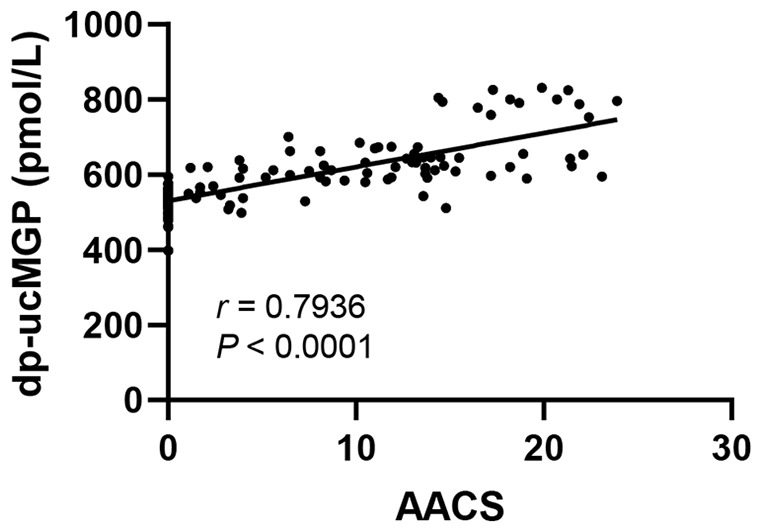
Correlation analyses of serum dp-ucMGP with abdominal aortic calcification score in PD patients.

To further analysis the correlation of serum dp-ucMGP, AACS, and clinical parameters, we performed the correlation analysis. As shown in Table 3 and Figure 3, the serum dp-ucMGP levels was positively associated with AACS (*r* = 0.794, *p* < 0.0001; Figure 2), BMI (*r* = 0.238), dialysis vintage (*r* = 0.312), serum TG (*r* = 0.162), phosphate (*r* = 0.037), Ca-P product (*r* = 0.192), ALP (*r* = 0.282), iPTH (*r* = 0.215), Cr (*r* = 0.293), and β2-MG (*r* = 0.262). Moreover, the serum dp-ucMGP levels was negatively correlated with RRF (*r* = −0.357).

**Table 3.** Correlation between serum dp-ucMGP, AACS and clinical characteristics.

Parameters	dp-ucMGP (ng/L)		AACS	
	*r*	*P*	*r*	*p*
Age, year	0.133	0.617	0.438	<0.01
BMI, kg/m^2^	0.238	0.024	0.216	0.047
Dialysis vintage, month	0.312	<0.01	0.179	0.172
Hb, g/L	0.039	0.572	0.028	0.249
ALB g/L	−0.14	0.296	−0.152	0.027
TG, mmol/L	0.162	0.029	0.241	0.027
TC, mmol/L	0.203	0.091	0.072	0.521
HDL, mmol/L	0.071	0.147	0.116	0.341
LDL, mmol/L	−0.152	0.316	−0.209	0.374
FBG, mmol/L	0.036	0.536	0.153	0.112
HbA1c	−0.021	0.281	0.029	0.729
Corrected Ca, mmol/L	0.051	0.593	0.271	0.046
P, mmol/L	0.037	0.025	0.151	0.562
Ca-P product, mmol^2^/L^2^	0.192	0.034	0.174	0.316
25(OH)D, µg/L	−0.137	0.682	0.359	0.503
ALP, U/L	0.282	<0.01	0.242	0.034
iPTH, ng/L	0.215	<0.01	0.271	0.025
Cr, mmol/L	0.293	<0.01	0.073	0.228
β2-MG, mg/L	0.262	<0.01	0.127	0.238
UA, mmol/L	0.136	0.259	0.263	0.158
hs-CRP, mg/L	0.028	0.651	0.437	<0.01
Kt/V urea, per week	0.017	0.122	0.026	0.103
RRF, mL/min	−0.357	<0.01	−0.239	<0.01
dp-ucMGP, pmol/L	–	–	0.794	<0.0001
AACS	0.794	<0.0001	–	–

All abbreviations are as defined in Table 1.

The AAC score was positively associated with age (*r* = 0.438), BMI (*r* = 0.216), serum TG (*r* = 0.241), corrected calcium (*r* = 0.271), ALP (*r* = 0.242), iPTH (*r* = 0.271), and hs-CRP (*r* = 0.437). Meanwhile, the AAC score was negatively associated with serum ALB (*r* = −0.152) and RRF (*r* = −0.239).

### Multivariate logistic regression analysis for risk factors of AAC in PD patients

Logistic regression analyses were performed to identify the determinants of vascular calcification in PD patients (Table 4). The variables related to vascular calcification in Table 2 were included in the logistic regression analysis. Results showed that age, dialysis vintage, Ca-P product, ALP, iPTH, hs-CRP, and dp-ucMGP (OR = 3.515) were associated with abdominal aortic calcification in PD patients.

**Table 4.** Regression analyses for abdominal aortic calcification in PD patients.

Variables	B	SE	Wald χ^2^	*p*	*OR*	95% CI
Age, year	1.035	0.427	5.471	0.031	2.815	1.518–4.786
Dialysis vintage, month	0.426	0.347	5.637	0.019	1.531	1.133–3.995
Ca-P product, mmol^2^/L^2^	0.403	0.406	5.889	0.015	1.496	1.248–4.040
ALP, U/L	0.339	0.312	7.563	0.001	1.404	1.101–3.555
iPTH, ng/L	0.691	0.453	5.359	0.042	1.996	1.590–5.315
hs-CRP, mg/L	1.018	0.445	7.140	0.006	2.768	1.442–6.250
dp-ucMGP, pmol/L	1.257	0.471	9.492	0.002	3.515	1.580–7.820

All abbreviations are as defined in Table 1.

**Figure 4.**
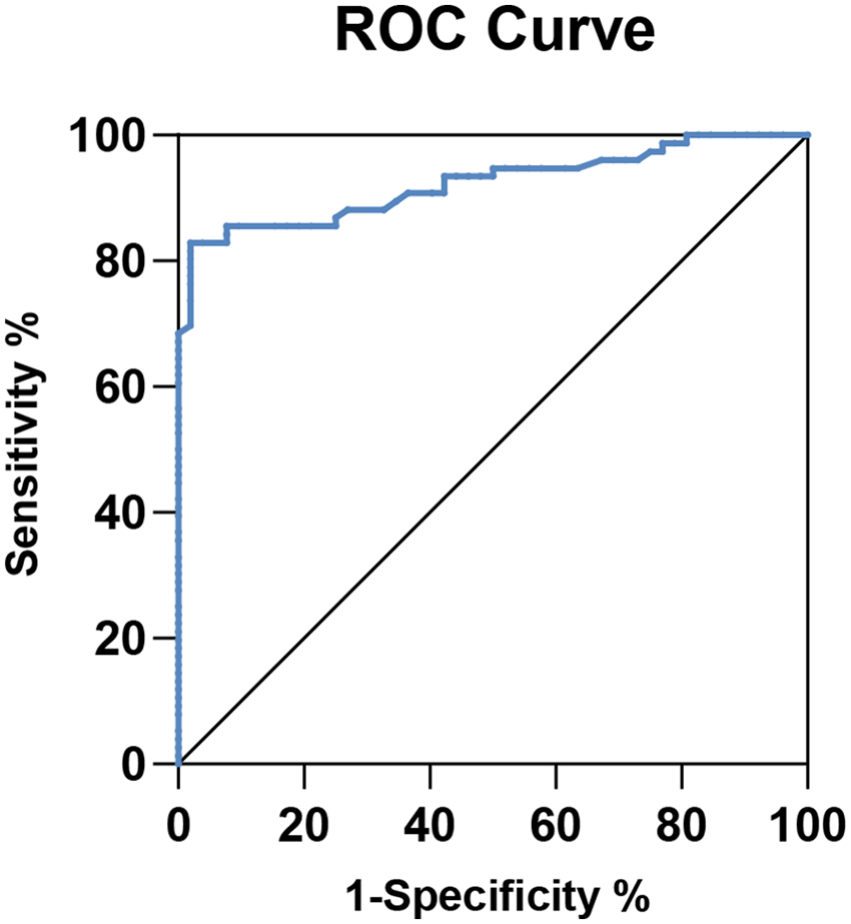
Receiver operating characteristic curves to predict AAC in PD patients.

According to the ACC score, a curve with (1-specificity) as the horizontal axis and sensitivity as the vertical axis was drawn to obtain the ROC curve, as shown in Figure 4. The cutoff point of dp-ucMGP to predict AAC in PD patients was 565.4 pmol/L, the area under the ROC curve was 0.9227, with a sensitivity of 85.53%, a specificity of 92.31%, a positive predictive value of 94.20% and a negative predictive value of 81.35%.

## Discussion

In this study, serum dp-ucMGP levels were positively associated with the AAC score by abdominal lateral X-rays in PD patients. In addition, serum dp-ucMGP levels was the independent predictor of vascular calcification in PD patients.

The levels of serum dp-ucMGP among health controls in our study were comparable to the data published by others, whereas serum dp-ucMGP levels in PD patients were lower than those in other studies.^8,14,20–22^ The different study population and platform for measuring dp-ucMGP may contribute to these differences. In keeping with results reported by others,^8,18^ we found that PD patients exhibited markedly elevated serum dp-ucMGP levels compared with the healthy controls. Next, our findings confirmed that the serum dp-ucMGP levels were also significantly higher in PD patients with AAC than PD patients without AAC. Schurgers et al. found that circulating dp-ucMGP was positively correlated with aortic calcification in CKD patients.^8^ In agreement with these results, our present study found that the serum dp-ucMGP levels increased as the AAC worsened, indicating the positive association of AAC severity with circulating dp-ucMGP levels in PD patients, which was further verified by correlation analysis. The area under the ROC curve of dp-ucMGP is 0.9227. And the sensitivity and the specificity are 85.53% and 92.31%, respectively, and the positive predictive value is 94.20% and the negative predictive value is 81.35%, when the cut-off point is 565.4 pmol/L, indicating that the level of serum dp-ucMGP has a good reference value for evaluating the degree of vascular calcification. In the Caucasian study, Griffin et al. demonstrated that an optimal cut-off for identifying participants with DKD was a plasma dp-ucMGP concentration exceeding 557 pmol/L.^21^ This threshold yielded an AUC of 0.842, with a diagnostic sensitivity of 67.0% and a diagnostic specificity of 91%. However, this study did not assess the capability of plasma dp-ucMGP to identify DKD participants with VC.

Emerging evidence has suggested that vitamin K is a vital regulator of cardiovascular calcification through carboxylation of MGP.^23–25^ Dp-ucMGP, the precursor of MGP, is a widely accepted biomarker of vitamin K deficiency, correlated with more ectopic calcification. Several randomized clinical trials have examined the possible effect of the vitamin K supplementation on clinical outcomes. Of note, most of these trials have been conducted in pre-dialysis CKD and/or HD patients.^23–26^ These clinical trials showed that oral administration of vitamin K2 improved vitamin K status in patients undergoing HD, whereas failed to withhold vascular calcification progression.^23,24,26^ However, the Valkyrie Study reported that long-term vitamin K2 administration at pharmacologic doses does not normalize systemic dp-ucMGP levels.^25^ Although the data has not published yet, Roumeliotis et al.^27^ is performing a prospective, randomized, open label, placebo-controlled trial, evaluating the effect of vitamin K2 supplementation on VC, cardiovascular events, and calcium/phosphorus metabolism in ESRD patients undergoing PD. Besides, dp-ucMGP was also identified as a survival marker, with lower levels heralding a better prognosis and decreased vascular calcification in previous studies.^8,14,28^ In agreement with previous studies conducted in cohorts of CKD, our results demonstrated that dp-ucMGP was negatively associated with residual renal function.^28^

Vascular calcification is a complex process involving many factors. Until now, risk factors for VC, including older age, longer dialysis vintage, diabetes, hypertension, dyslipidemia, and smoking are confirmed both in CKD cohorts and non-CKD cohorts.^29,30^ Moreover, hyperphosphatemia, elevated calcium-phosphate product, and hyperparathyroidism are well-recognized predictors of VC in dialysis patients. Consistently, our results showed that the PD-AAC group had higher BMI, longer dialysis vintage, serum corrected calcium, phosphate, calcium phosphorus product, iPTH, UA, and hs-CRP, but lower serum ALB than the PD-nonAAC group. We also found that the AAC score was positively associated with age, BMI, corrected calcium, ALP, and iPTH, while negatively correlated with ALB. The AAC risk factors identified in our study included age, dialysis vintage, corrected calcium, Ca-P product, ALP, iPTH, hs-CRP, and dp-ucMGP. These findings indicated that increased circulating calcium load in PD patients is associated with elevated vascular calcification. Multiple studies suggested that MGP acts as a calcification-inhibitor by inhibition of calcium-phosphate precipitation, the formation of matrix vesicles, the formation of apoptotic bodies, and trans-differentiation of vascular smooth muscle cells.^31^ Consistently, we also compared the correlation between dp-ucMGP and several clinical parameters and found that serum dp-ucMGP was significantly associated with phosphate, calcium phosphorus product, ALP, and iPTH.

We found that 59.4% PD patients suffered from abdominal aortic calcification, in accordance with published data in Asia populatKid Int Repion.^32^ Compared to hemodialysis, peritoneal dialysis patients have better residual renal function and electrolyte balance. Besides, Ca, phosphate, iPTH, and bone turnover seem to play a lesser role in vascular calcification among PD patients compared with HD patients.^33^ Thereby, the incidence of calcification in the membrane PD group is lower than that in hemodialysis group.^34,35^ Additionally, the association of Ca, phosphate, and iPTH with mortality among PD patients were somewhat different from those among HD patients.^36,37^ The Ca-P balance between hemodialysis and peritoneal dialysis needs to be further explored, as well as the association between vascular calcification and dialysis modality.

There are still several limitations in this study. First, the sample size is small, and all samples are from the same region. There might be some selection bias, and the results from various areas and different samples might differ. Second, this study is cross-sectional, and we cannot figure out the causal relationship between AAC and dp-ucMGP. Third, due to lack of follow-up information, the relationship between AAC progression and dp-ucMGP remains unknown. Larger multicenter studies are needed in the future.

In conclusion, our study implies the positive association between serum dp-ucMGP and AAC score in PD patients. Higher serum dp-ucMGP level is independently associated with AAC in PD patients. These findings indicate that dp-ucMGP could be used as a potential indicator of VC for further investigation.

## Supplemental Material

sj-pdf-1-jao-10.1177_03913988241293980 – Supplemental material for Correlation between circulating dephosphorylated uncarboxylated matrix Gla protein and vascular calcification in peritoneal dialysis patients Supplemental material, sj-pdf-1-jao-10.1177_03913988241293980 for Correlation between circulating dephosphorylated uncarboxylated matrix Gla protein and vascular calcification in peritoneal dialysis patients by Liman Mao, Haole Huang, Meiyang Zhou and Canxin Zhou in The International Journal of Artificial Organs
