# Supplementary material for: Correlation between circulating dephosphorylated uncarboxylated matrix Gla protein and vascular calcification in peritoneal dialysis patients
Source: Int J Artif Organs. 2024 Oct 31;47(12):885–93. doi: 10.1177/03913988241293980 (PMC11613520; doi:10.1177/03913988241293980)
Supplement: sj-pdf-1-jao-10.1177_03913988241293980 – Supplemental material for Correlation between circulating dephosphorylated uncarboxylated matrix Gla protein and vascular calcification in peritoneal dialysis patients [file sj-pdf-1-jao-10.1177_03913988241293980.pdf]

Peritoneal dialysis (PD) procedure

All PD patients were dialyzed with 1.5% to 2.5% low calcium glucose dual system PD solution (Dianeal®, Baxter, China), which was infused intraperitoneally with 2L each time, 3~4 times per day. Continuous ambulatory peritoneal dialysis was performed with 3 bags left in the abdomen for 4 hours during the day and the last bag left in the abdomen at night.

Dp-ucMGP enzyme-linked immunosorbent assay (ELISA) protocol

This kit was based on sandwich enzyme-linked immune-sorbent assay technology. Anti dp-ucMGP antibody was pre-coated onto the 96-well plate. The biotin conjugated anti dp-ucMGP antibody was used as the detection antibody. The standards and pilot samples were added to the wells subsequently. After incubation, unbound conjugates were removed by wash buffer. Then, biotinylated detection antibody was added to bind with dp-ucMGP conjugated on coated antibody. After washing off unbound conjugates, HRP-Streptavidin was added. After a third washing, TMB substrates were added to visualize HRP enzymatic reaction. TMB was catalyzed by HRP to produce a blue color product that turned yellow after adding a stop solution. Read the O.D. absorbance at 450nm in a microplate reader. The concentration of dp-ucMGP in the sample was calculated by drawing a standard curve. The concentration of the target substance is proportional to the OD450 value. The intra- and inter-assay variability values were 5.0% and 5.0%, respectively. The ELISA kit measuring range was between 78.125 and 5000 pg/mL.

Abdominal aortic calcification (AAC) scoring criteria

AACS was computed by a validated scoring system to grade individual calcified lesions at both the anterior and posterior walls of the aorta parallel to vertebrae level 1 to level 4 based on abdominal lateral X-rays, which was first proposed by Kauppila[23]. 0 points: no calcification deposition; 1 point: calcification deposition < 1/3 of the length of the arterial wall; 2 points: calcification deposition affects 1/3 to 2/3 of the length of the arterial wall; 3 points: calcification deposition ⩾ 2/3 of the length of the arterial wall. Finally, add up the scores obtained from the anterior and posterior

walls of each vertebral body. The horizontal score range for each vertebral body of each patient is 0-6 points, and the total range of AACs is 0-24 points.
